# Neuromuscular Fatigue and Metabolic Stress during the 15 Minutes of Rest after Carrying Out a Bench Press Exercise Protocol

**DOI:** 10.3390/biology11101435

**Published:** 2022-09-30

**Authors:** Juan Hernández-Lougedo, Juan Ramón Heredia-Elvar, Luis Maicas-Pérez, Ana María Cañuelo-Márquez, Manuel Rozalén-Bustín, Fernando de Jesús Franco, Manuel Vicente Garnacho-Castaño, Pablo García-Fernández, José Luis Maté-Muñoz

**Affiliations:** 1 Department of Physical Activity and Sports Science, Alfonso X El Sabio University, 28691 Madrid, Spain; 2 Department of Physiotherapy, Alfonso X El Sabio University, 28691 Madrid, Spain; 3 Faculty of Health Sciences, International University of La Rioja, 26006 Logroño, Spain; 4 Campus Docent Sant Joan de Déu, University of Barcelona, 08034 Barcelona, Spain; 5 Department of Radiology, Rehabilitation and Physiotherapy, Complutense University of Madrid, 28040 Madrid, Spain; 6 IdISSC, Instituto de Investigación Sanitaria del Hospital Clínico San Carlos, 28040 Madrid, Spain

**Keywords:** strength, sport performance, human performance, velocity, external load, internal load, training, repetitions

## Abstract

**Simple Summary:**

Velocity loss can help to determine neuromuscular fatigue after carrying out an exercise protocol with a load that is initially moved at a mean propulsive velocity of 1 m·s^−1^. Few studies have measured the time required to recover the pre-exercise velocity values after performing an exercise, with the aim of knowing the optimal recovery times. Therefore, the aim of this study was to analyse muscular fatigue and metabolic stress during the 15 min that follow the execution of a bench press exercise protocol. The results show that mean propulsive velocity was recovered from minute 10 of rest; however, the blood lactate levels were still high (>5 mmol·L^−1^). Therefore, although the capacity to apply a maximum mean propulsive velocity peak was recovered, the same could not be said for the capacity to maintain those strength levels to reproduce another exercise protocol involving the same muscle groups. Moreover, analysing the velocities and metabolic stress before and after the exercise protocol by different strength level groups, similar results were obtained.

**Abstract:**

Background: Velocity loss (VL) at 1 m·s^−1^ can help to determine neuromuscular fatigue after performing an exercise protocol. The aim of this study was to analyse muscle fatigue and metabolic stress during the 15 min that follow the execution of a bench press (BP) exercise protocol. Methods: Forty-four healthy male students of sports science performed two exercise sessions separated by one week of rest. In the first week, the participants carried out a test with progressive loads in the (BP) exercise until reaching the one-repetition maximum (1RM) in order to obtain the load–velocity relationship of each participant. In the second week, each participant conducted three BP exercise sets at an intensity of 70% of 1RM, determining this load through the mean propulsive velocity (MPV) obtained from the individual load–velocity relationship, with the participants performing the maximum number of repetitions (MNR) to muscle failure. Two minutes of rest were allocated between sets. MPV at 1 m·s^−1^ and blood lactate concentrations were recorded before executing the exercise and at minute 0, 5, 10 and 15 after performing the exercise protocol. Results: A two-factor repeated measures ANOVA was performed. MPV at 1 m·s^−1^ in minute 0 post-exercise was −33.3% (*p* < 0.05), whereas in minute 10 and 15 post-exercise, it was ≈−9% (*p* > 0.05). Regarding the blood lactate levels, significant differences were observed in all measurements before and after the exercise protocol (*p* < 0.001), obtaining ≈7 mmol·L^−1^ at minute 10 post-exercise and 4.3 mmol·L^−1^ after 15 min of recovery. Conclusions: MPV with medium or moderate loads shows a certain recovery from minute 10 of rest. However, the blood lactate levels are still high (>5 mmol·L^−1^). Therefore, although there seem to be certain conditions to reach a similar maximum MPV peak, the residual fatigue at the neuromuscular level and the non-recovery of metabolic homeostasis would hinder the reproduction of these protocols, both at the level of applied stimulus and from a methodological perspective, since a long recovery time would be required between sets and exercises.

## 1. Introduction

The current literature suggests that greater muscle strength improves the strength-time characteristics (rate of force development (RFD) or external mechanical power), being the basis of many physical attributes and a potential influential factor in the improvement of the general athletic performance of both athletes and the general population [1]. Moreover, the improvement of muscle strength has an impact on potentiation and on the decrease in the injury rate [1]. However, being able to define the adequate stimulus, objectively quantifying the real load in strength training, is one of the main problems that exercise professionals face nowadays [2,3]. In the design of strength programmes, several variables have been identified, such as exercise intensity, volume (number of sets and repetitions), rest duration and the type and order of the exercises [4,5]. Considering these variables, as a general guideline, recommendations have been established for resistance training with different progressions for different fitness levels. For novices, these recommendations establish relative intensities equivalent to 60–70% of one–repetition maximum (1RM), performing between one and three sets of 8–12 repetitions each, setting a rest interval of 2–3 min between set [6]. For intermediate fitness levels, relative intensities were equivalent to 70–80% of 1RM, performing multiple sets of 6–12 repetitions with 1–2 min rest between sets [6]. Furthermore, when the accurate measurement of execution velocity in isoinertial strength exercise was developed, this variable became very important in strength training programmes [2,7]. Velocity-based training has been proposed as an effective and objective method to quantify training intensity, since the real load can be controlled (%1RM), measuring the velocity of the first repetition [2,8,9]. Moreover, velocity loss (VL) during the training set could be used as a criterion to control the number of repetitions performed [10,11], which may allow relating, with great precision, the percentage of repetitions completed from a certain VL [12]. VL throughout the sets also influences the duration of the rests between sets. A recent study has determined that 2 min of rest between sets (3 x performing the maximum number of repetitions to muscle failure (MNR) at 70% mean propulsive velocity (MPV)) is insufficient to recover the velocity of the first set [13]. In addition, VL can help to determine neuromuscular fatigue. Sánchez-Medina and González-Badillo (2011) [10] described a method to calculate the percentage of VL after performing an exercise protocol with a load that is initially moved at an MPV of 1 m·s^−1^, reporting a strong association between the magnitude of VL and the degree of fatigue. Subsequent studies have also used the pre–post exercise change in velocity attained against MVP at 1 m·s^−1^ load for the measurement of muscle fatigue in both bench press and squat exercises. [14,15,16,17]. By measuring this mechanical variable, they have been able to demonstrate that the time needed for recovery may significantly increase as the repetition number approaches muscle failure. Recovery time is important as it can influence subsequent training sessions [15,16]. However, few studies have measured the time that VL can be maintained or the moment at which the pre-exercise velocity values are recovered within the same training session. In a recent study, Maté Muñoz et al. (2022) [18] measured neuromuscular fatigue in an endurance session of functional fitness training (FFTendurance) and in a strength session of functional fitness training (FFTstrength) through VL, reporting that this velocity was recovered after 15 min of rest. Although the velocity values of FFTendurance were recovered after 15 min of rest, in the FFTstrength session, velocity was only recovered in the 1 m·s^−1^ tests in squat, whereas velocity in the 1 m·s^−1^ tests in military press exercise decreased by 7%. However, to the best of our knowledge, no studies have measured this recovery in strength training protocols with bench press (BP), squat, deadlift, etc. If this VL is related to a certain degree of fatigue, recovering the initial velocity values could help to determine the recovery time required to perform another exercise protocol reaching the same velocity in the first repetition (i.e., same %1RM) as in the pre-exercise. In other words, the pre–post exercise change in velocity attained against MVP at 1 m·s^−1^ load after performing an exercise protocol, would allow athletes to be in a better neuromuscular condition to perform a new training session on the same day.

In this regard, another recent study aimed to identify recovery times after performing the sprints, evaluated the effects of an experimental short-time warm-up consisting of a small number of intermittent high-intensity sprints (three maximum sprints over 60 m with 120 s of recovery between sprints) on explosive muscle strength performance in soccer players [19]. The data indicated that only 330 s (5 min and 30 s) after the last sprint, the countermovement jump performance recovered to baseline levels [19]. These results are in line with another study documenting that performance was optimal with recovery times between 7 and 10 min for intensities between 60 and 85% of 1RM [20].

Therefore, the aim of this study was to analyse muscle fatigue and metabolic stress during the 15 min that follow the execution of a BP exercise protocol. Our hypothesis proposes that 15 min of rest after performing a BP exercise protocol may be necessary to begin finding a certain recovery in the capacity to apply strength (maintaining MPV levels) with respect to the pre-exercise levels.

## 2. Materials and Methods

### 2.1. Study Design 

This cross-sectional study consisted of performing two exercise sessions separated by one week of rest. In the first week, with the aim of obtaining the load–velocity relationship of each participant, a BP exercise test with progressive loads was carried out until reaching the 1RM. In the second week, each participant conducted three sets of the BP exercise at an intensity of 70% of 1RM, performing the MNR, with two minutes of rest between sets. To control the effects of the circadian rhythms [21], the two sessions of the BP exercise protocol were carried out with a temporal interval of no more than two hours (± 2 h) on the same day of the week. All exercise sessions were performed in the exercise physiology laboratory of the university. The ambient temperature ranged between 18º and 22º C, and the relative humidity ranged between 40 and 65%. 

### 2.2. Participants

**Figure 1:**
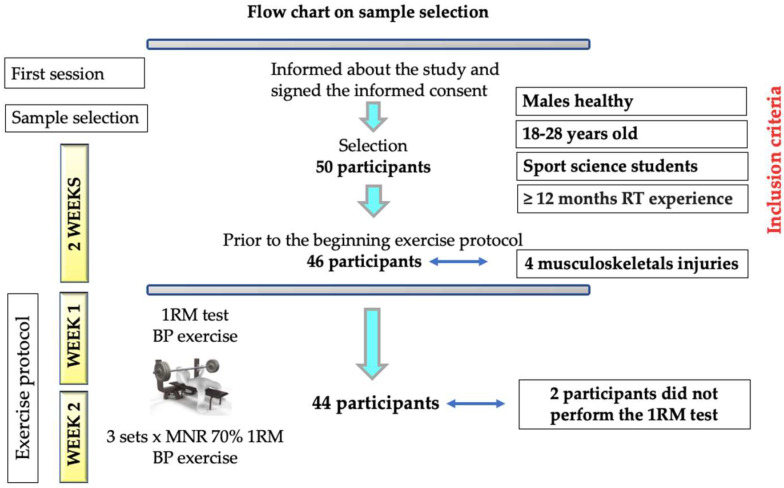
Flow chart on sample selection. RT = resistance training, BP = bench press, 1RM = one-repetition maximum test. MNR = maximum number of repetitions.

Forty-four healthy male students of sports science (age = 22.7 ± 3.6 years, body weight = 78.0 ± 10.9 kg, height = 1.8 ± 0.1 m, body mass index = 24.3 ± 2.7 kg·m^2−1^, 1RM = 82.6 ± 17.1 kg, and relative strength ratio (RSR) (obtained from 1RM strength/body mass) = 1.07 ± 0.22), participated in the study. These participants were divided into 3 groups based on their RSR— (1) High RSR (>1.25; *n* = 11), (2) Medium RSR (between 1.05 and 1.25; *n* = 14), (3) Low RSR (<1.05; *n* = 19)—based on the cut-off points of a recent study [3]. The participants knew how to perform the BP exercise correctly. However, one week before initiating the study, two sessions were carried out to allow the participants to familiarise with the BP exercise, with a period of 48 h between them. All participants attended these familiarisation sessions. As inclusion criteria, participants had to be healthy males aged 18–28 years and sports science students with at least 12 months of strength training experience. The exclusion criteria were to present musculoskeletal injuries or cardiorespiratory or metabolic pathologies that could limit their performance. Likewise, during the study, none of the participants used drugs or food supplements. They were asked not to eat any food two hours before the test, although they were allowed to drink water. All participants were requested to avoid performing physical exercise the day before. The GRANMO statistical calculator was used, using the standard deviation determined in a previous pilot study with 10 sports science students assuming an alpha error of 0.05, a power of 0.85 and a percentage of loss of 12%. With these data, a sample size of 50 participants per intervention group was estimated. All participants were initially informed about the study in a previous session, and they voluntarily signed the informed consent (Figure 1). The study design was presented and approved by the ethics committee of the University, following the principles of the Declaration of Helsinki [22]. 

### 2.3. Procedures

#### 2.3.1. One-Repetition Maximum (1RM) Test

**Figure 2:**
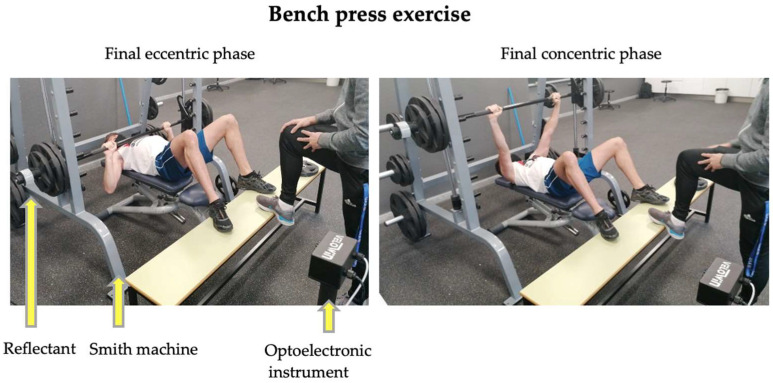
Bench press exercise performed by one of the study participants.

A test with progressive loads was performed in the BP exercise until reaching the 1RM (1RM test), based on the description of the BP exercise protocol of a previous study [2], obtaining the individual load–velocity relationship of each participant. Before conducting the 1RM test, the participants performed a warm-up consisting of 5 min of low-intensity running and 5 min of joint mobility and dynamic stretching exercises, which was followed by one set of 10 repetitions of BP with a fixed load of 10 kg, and one set of 5 repetitions of BP with a fixed load of 20 kg. The technical execution of the BP exercise was as follows: in the supine position on the bench, with hips and knees bent, placing the feet on the bench, and the arms opened slightly beyond shoulder width. The bar was lowered slowly and in a controlled manner until reaching the chest, right above the intermamillary line, holding it for 2 s on the chest to avoid the bouncing effect and to improve the reliability of the repetitions [23] (Figure 2). A researcher counted 2 s of stopping between the eccentric and concentric phases and then gave the verbal order to execute the concentric phase. The participants were asked not to lift their shoulders or trunk from the bench and avoid bouncing during the concentric phase, which was required to be executed at the maximum possible velocity. 

#### 2.3.2. 3 × Maximum Number of Repetitions (MNR) Exercise Protocol

One week after the 1RM test, the MNR protocol was carried out. This consisted of the execution of three sets of the BP exercise against 70% 1RM, determining this load through the MPV obtained from the individual load–velocity relationship. In each of the three sets, the objective was to perform the MNR. The rest time between sets was 2 min. This exercise protocol is based on the American College of Sports Medicine strength training recommendations for novice and intermediate levels [6].

#### 2.3.3. Blood Lactate Concentrations

In order to measure metabolic stress, before the warm-up and at minute 0, 5, 10 and 15 after the MNR, capillary blood samples (5 μL) were extracted from each participant at the index finger to obtain the lactate concentrations. 

#### 2.3.4. Mechanical Fatigue Test

To calculate the mechanical fatigue induced in the 3 × MNR exercise protocol, we used the change percentage of the pre–post exercise with an individual load that could be lifted at ≈1 m·s^−1^ of MPV during the propulsive phase, which is defined as the portion of the concentric phase during which the acceleration of the bar is ≥9.81 m·s^−2^ (MPV at 1 m·s^−1^ Test) of a BP. This mechanical fatigue test is based on the one conducted by Sánchez Medina and González-Badillo (2011) [10]. It was decided to measure the value of 1 m·s^−1^, as it is a sufficiently high velocity, obtained in medium or moderate loads (45–50% RM in BP), which allows providing a good expression of the effect of the load on velocity; additionally, this load is well tolerated and relatively easy to move. The test began with a weight of 10 kg, which was increased by 1.25–5 kg, performing 3 repetitions with each load, resting 3 min between loads until reaching the individual load that can be moved at a velocity of 1 m·s^−1^. All repetitions were conducted at maximum velocity. The velocity of execution of each repetition was recorded with an optoelectronic instrument. The velocities reported in the present study correspond to the mean velocity of the propulsive phase for each repetition. Mean propulsive values are preferable to mean concentric values, as this avoids underestimating an individual’s true neuromuscular potential when lifting light and medium loads [24]. Once we obtained the individualised load at which a velocity of 1 m·s^−1^ was reached, 3 repetitions of BP were executed before and at minute 0, 5, 10 and 15 after the 3 × MNR exercise protocol.

#### 2.3.5. Measurement Equipment

A Smith machine with guide rod and multipower system (Matrix, Chácara Alvorada, Brazil) was used for the different BP exercise protocols. Since the two ends of the bar are fixed, only the vertical movement of the bar was allowed. The discs used were 1.25, 2.5, 5, 10 and 20 kg (Matrix). To estimate the velocity of execution of each repetition, a previously validated optoelectronic instrument was used [25]. The sampling frequency of the optoelectronic instrument was 500 Hz (Velowin v.1.7.232, Instrumentos y Tecnología Deportiva; Murcia, Spain). The instrument was calibrated following the manufacturer’s instructions. The calculations of MPV were automatically performed with the algorithms of each software (Velowin v.1.7.232). In addition, to obtain the capillary blood lactate samples, a previously validated and calibrated portable analyser was employed (Lactate Pro 2 LT-1710, Arkray Factory Inc., KDK Corporation, Siga, Japan) [26,27].

#### 2.3.6. Analysed Variables

The MPV of each repetition of each set was measured in the different tests conducted in BP, as well as the MPV at 1 m·s^−1^ in the mechanical fatigue test. Moreover, in the 3 × MNR exercise protocol, the following parameters were measured: blood lactate concentrations before and at minute 0, 5, 10 and 15 after the exercise; MPV of the best (fastest) repetition of each set (MPV_rep_ Best); MPV attained at the last repetition of each set (MPV_rep_ Last); and loss of MPV (% loss MPV Set), defined as (MPV_rep_ Last − MPV_rep_ Best)/MPV_rep_ Best × 100.

### 2.4. Statistical Analysis

The Shapiro-Wilk test was initially carried out to determine the normality of the variables. Second-order polynomials were used to establish the load–velocity relationship for each participant in the progressive load test up to 1RM. To analyse the different variables of the 3 × MNR exercise protocol in the entire sample of participants, a single-factor repeated measures ANOVA was performed, comparing it with Mauchley’s sphericity test. When the sphericity test was rejected, the univariate F-statistic was used, adjusting it with the Greenhouse–Geisser correction index. When significant differences were found between measurements, Bonferroni’s post hoc test was applied. To analyse the BP exercise protocol based on the different strength levels, a two-factor repeated measures ANOVA was performed for the time factor, applying Levene’s test to assess the homogeneity of variances. Therefore, it is considered that there is an inter-subject factor with 3 groups by level (High RSR, Medium RSR, Low RSR) and an intra-subject factor with the variable “time” in 5 levels (pre-exercise, minute 0 post-exercise, minute 5 post-exercise, minute 10 post-exercise, minute 15 post-exercise) (3 groups × 5 times), also observing the effect of the interaction and applying Bonferroni’s post hoc index for pairwise comparison. Moreover, the statistical power (SP) of the data was determined, as well as the effect size, known as partial eta squared (η_p_^2^), categorising the magnitude of the difference as trivial (η_p_^2^ ≤ 0.01), small (0.01 ≤ η_p_^2^ < 0.06), moderate (0.06 ≤ η_p_^2^ < 0.14), or large (η_p_^2^ ≥ 0.14) [28]. Furthermore, linear regression and correlation models were used to establish a relationship between the blood lactate concentrations and the loss percentage of MPV at 1 m·s^−1^ in each of the measured time points. All data are expressed as means, standard deviations, 95% confidence intervals and coefficient of variation. The level of significance was set to *p* < 0.05. All statistical tests were performed using the statistical package SPSS version 25.0 (SPSS, Chicago, IL, USA). 

## 3. Results

**Figure 3:**
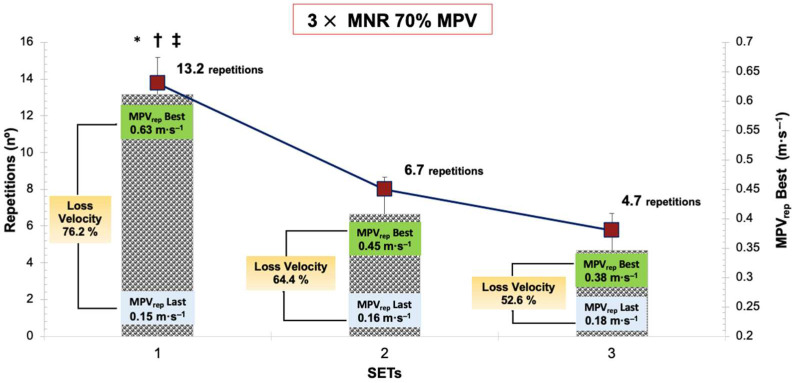
Comparison of the repetitions performed in the three sets, with the MPV of the best repetition and velocity loss percentage between the MPV of the best repetition and the MPV of the last repetition. Rep = repetition; MPV = Mean Propulsive Velocity; MRN = Maximum Number of Repetitions; * Repetitions = significant difference between all sets (*p* < 0.05); † MPV_rep_ Best = significant difference between all sets (*p* < 0.05); ‡ % loss MPV = significant difference between all sets (*p* < 0.05).

Figure 3 presents the different variables measured in the BP exercise protocol.

**Figure 4:**
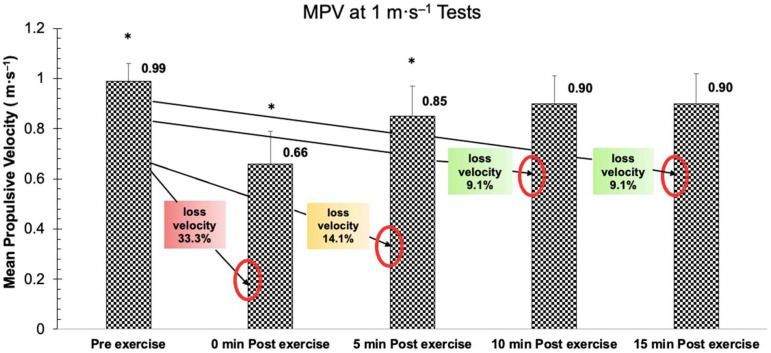
MPV at 1 m·s^−1^ before the BP exercise protocol and at minute 0, 5, 10 and 15 after the exercise. * = significant difference between all sets (*p* < 0.05).

The obtained data of the MPV at 1 m·s^−1^ test before and after the BP exercise protocol show significant differences between all measurements (*F* (4,43) = 169.885, *p* < 0.001, η_p_^2^= 0.798, SP = 1.000), except for the MPV at minute 10 and 15 after the exercise (*p* > 0.05) (Figure 4). 

**Figure 5:**
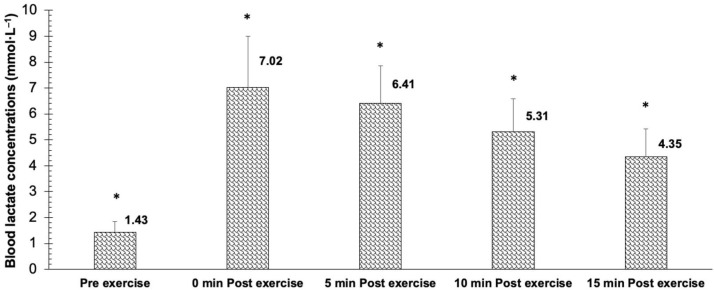
Blood lactate concentrations before and the BP exercise protocol and at minute 0, 5, 10 and 15 after the exercise. * = significant difference between all sets (*p* < 0.05).

Regarding the blood lactate levels, significant differences were identified between all measurements before and after the BP exercise protocol (*F* (4,43) = 222.897, *p* < 0.001, η_p_^2^= 0.838, SP = 1.000) (Figure 5). 

Analysing the variables by strength level (Table 1), significant differences were found in MPV at 1 m·s^−1^ only in the “time” factor (*p* < 0.05). However, for the blood lactate concentrations, significant differences were obtained in three factors: time, group and time × group interaction (*p* < 0.05). The pairwise comparison through Bonferroni’s post hoc correction for the blood lactate concentrations of group × time showed significant differences between minute 0 post-exercise and minute 10 and 15 post-exercise in High RSR and Low RSR (*p* < 0.001, *p* < 0.001 and *p* = 0.002, *p* < 0.001, respectively), whereas in Medium RSR, there was statistical significance between minute 0 post-exercise and minutes 5, 10 and 15 post-exercise (*p* = 0.047, *p* < 0.001, *p* < 0.001, respectively). Blood lactate at minute 5 post-exercise showed statistically significant differences with minute 10 and 15 post-exercise (*p* < 0.001) in all strength levels. For High and Medium RSR, blood lactate maintained significant differences between minute 10 and minute 15 post-exercise (*p* < 0.001). In the analysis of time × group, significantly different blood lactate values were observed between High RSR and Low RSR in minute 0 and 10 post-exercise (*p* = 0.021, *p* = 0.41, respectively).

**Figure 6:**
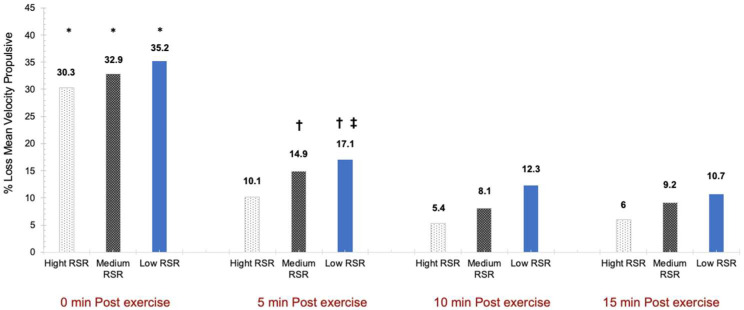
Percentage loss MPV at 1 m·s^−1^ at minute 0, minute 5, minute 10 and minute 15 compared to the pre-exercise after the BP exercise protocol. RSR = Relative Strength Ratio. * = significant difference of minutes 0 post-exercise with minutes 5, 10 and 15 post-exercise (*p* < 0.05). † = significant difference between minutes 5 and 10 post-exercise (*p* < 0.05). ‡ = significant difference between 5- and 15-min post-exercise (*p* < 0.05).

Analysing the VL percentage in the different post-exercise time points with respect to the pre-exercise for each strength level group, significant differences were observed for the “time” factor (*F* (3,41) = 151.072, *p* < 0.001, η_p_^2^= 0.787, SP = 1.000), whereas no differences were detected for the “group” factor (*F* (2,41) = 1.833, *p* = 0.173, η_p_^2^= 0.082, SP = 0.360) or in the effect of the time × group interaction (F (6,41) = 0.255, *p* = 0.928, η_p_^2^= 0.012, SP = 0.108) (Figure 6).

**Figure 7:**
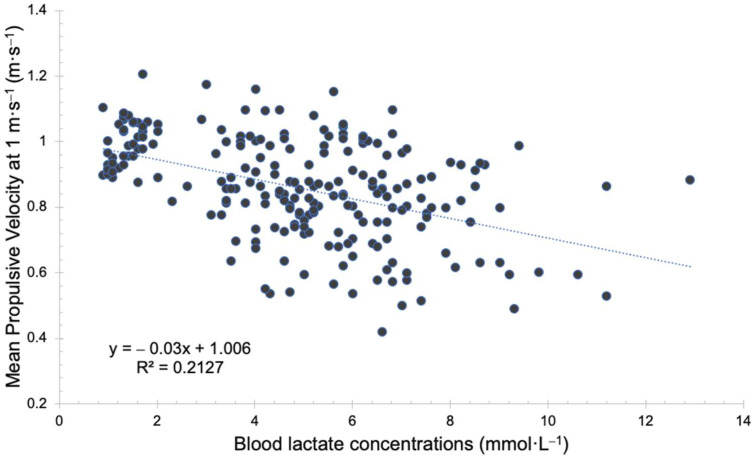
Linear regression between the blood lactate concentrations and MPV at 1 m·s^−1^ before the exercise protocol and at minute 0, 5, 10 and 15 after the BP exercise protocol of the 44 participants.

In the regression analysis conducted to establish an association between the blood lactate concentrations and the percentage loss of MPV with respect to pre-exercise, it was observed that such association was weak (*R*^2^ = 0.213, *p* < 0.001) (Figure 7), with a strong relation (*R* = 0.461, *p* < 0.001).

## 4. Discussion

The main finding of this study is that after carrying out an exercise protocol of three sets at 70% of 1RM at MNR (muscle failure) with 2 min of rest between sets, the capacity to reach a maximum MPV peak with medium or moderate loads seems to show a certain recovery from minute 10 of rest, being still ≈9% lower than the pre-exercise values. This fact must be analysed in relation to the existing alteration of metabolic homeostasis. At this time point, the lactate values were >5 mmol·L^−1^. At minute 15 post-exercise, the MPV values are practically similar to those at minute 10, with the blood lactate values being almost 1 mmol·L^−1^ lower. In this way, we could confirm our initial hypothesis. This could indicate different recovery curves, where the neuromuscular factor may recover faster to reach a maximum MPV peak with medium or moderate loads, whereas the metabolic factor would still require more time to approach the baseline levels, which could compromise the capacity to perform the MNR again in another protocol where the same muscle groups are involved. That is, this recovery of the maximum MPV peak, despite showing no statistically significant differences, should be considered, since this level of residual fatigue, linked to neuromuscular capacity itself, could be a potential limiting factor in the performance of subsequent tasks.

Analysing the VL at the end of the exercise protocol, differences are observed with other studies. In one of them, 61.2% was lost performing 3 × 8 at 80% 1RM at muscle failure [14]. In another study, performing 3 × 10 at 75% 1RM also at muscle failure, values similar to those reported in our study were obtained (≈0.88 m·s^−1^), and differences were found compared to the protocols that did not reach muscle failure (≈1 m·s^−1^) [16]. Then, 6 h after the exercise protocol, the initial values were recovered (≈0.98 1 m·s^−1^) [16].

Comparing these results with those of another study that measured the physiological responses of an FFT protocol up to 15 min of rest, it was observed that MPV at 1 m·s^−1^ was not recovered only in the military press test of FFTstrength [18]. However, in the fatigue test at 1 m·s^−1^ in squat of the FFTstrength and in the fatigue tests in military press and squat of FFTendurance, the pre-exercise values were recovered, despite obtaining values of ≈13 mmol·L^−1^ after 15 min of rest [18]. The high values of blood lactate compared to those obtained in this protocol (≈4 mmol·L^−1^) may be due to the different nature of the exercises and the time used to execute each exercise. However, in spite of these differences between the exercise protocols, the values of MPV at 1 m·s^−1^ are recovered between minute 10 and 15. Another study about FFT also observed a recovery of jump capacity after 20 min of rest [29]. Therefore, these results indicate that regardless of the metabolic stress obtained after a recovery period of 15–20 min, in different types of exercise, the mechanical variables that measure neuromuscular fatigue, such as MPV at 1 m·s^−1^ and jump height in a countermovement jump test, recover their initial values. This would be in line with the weak association between the blood lactate concentrations and the percentage of MPV in this study (*R*^2^ = 0.213). 

Thus, the results obtained in this study suggest that a minimum of 10 min of rest would be necessary to reach similar strength peaks in upper-limb push actions with medium or moderate loads, although this does not guarantee a “complete” recovery or the capacity to reproduce a similar performance level with these or other loads. This could be linked to the non-recovery of the pre-exercise lactate levels; in this sense, metabolic stress could be a barrier to completing the same number of repetitions that were carried out in each of the previous three sets. Fatigue was defined several decades ago as the transitory loss of the voluntary capacity to produce strength during exercise [30], which is caused by the integration of regulatory mechanisms at multiple biological levels [31]. This decrease in the capacity to apply strength could originate in several levels of the neural axis, in the motor cortex, between the spinal cord and the neuromuscular junction, in the muscle membrane and in metabolism [32]. After 10 min of rest, the voluntary activation of the muscle by part of the nervous system may recover the capacity to apply the maximum strength peak, thanks to the increase in efference of the upper motor centres towards the motoneurons, the decrease in the synaptic inhibition aimed at the motoneurons and the intrinsic adaptations in the motoneurons that make these progressively more reactive to synaptic excitation during exercise [33,34]. However, being able to maintain the strength levels during a full BP exercise protocol may not be possible due to the high metabolic stress. The increase in the lactate concentrations reduces the contractile capacity of the muscle [35] due to both the accumulation of hydrogen ions, which reduce the pH and generate metabolic acidosis, and the inhibition of phosphofructokinase [36]. Moreover, high-intensity and short-duration exercises have been related to factors that are rather associated with the appearance of peripheral fatigue [32], phosphagen depletion [37], accumulation of inorganic phosphate [38] and alteration of calcium levels [39]. Therefore, in this study, reproducing again the 3 sets at MNR with 70% 1RM could not be possible after 15 min of rest. In this regard, the analysis of the MNR performed in each of the sets of this study, together with the MPV of the best repetition of each set, shows that they are significantly lower with each subsequent set, and that it is not possible to reproduce the MNR in each of the sets with 2 min of rest. This fact has been reported in a recent study [13]. Therefore, the recovery time that must be allocated after executing an exercise protocol to subsequently execute another protocol with the same muscle group has been poorly studied. There are very generic propositions of recovery depending on whether fatigue is central or peripheral, whether the exercise is short or long, and whether exercise is of low or high intensity [32], although further studies are required to determine the optimal recovery times after performing an exercise protocol.

The analysis of the results differentiating the participants by their RSR shows that no differences were obtained between groups in the MPV at 1 m·s^−1^ of the fatigue test. However, in the blood lactate concentrations, there were significantly higher levels in High RSR with respect to Low RSR for minute 0 and 10 post-exercise. This could be due to the fact that a heavier load was lifted for the same relative intensity in High RSR. However, at minute 15 post-exercise, the blood lactate levels were similar among all strength levels (≈4.5 mmol·L^−1^). On the other hand, in relation to the velocity loss percentage, there were no significant differences between the different RSR during the different post-exercise measurements. Nevertheless, percentage-wise, the High RSR group showed lower velocity loss throughout the entire recovery time. This suggests that MPV recovery can be different depending on the strength level. Therefore, based on the results obtained in this study, and considering the scarce literature found in this regard, it is necessary to conduct further studies that delve into the topic of recovery after exercise.

As the aim is to maximise the performance of a training session where a BP exercise protocol is performed at muscle failure (3 × MNR at 70% 1RM), at least 10 min of rest would be needed to recover the ability to generate similar MPV values to those obtained in the pre-exercise. However, if wanted to perform another exercise protocol with the same muscle groups, because lactate concentrations are still high (>4 mmol·L^−1^), it would be necessary to use different strategies to optimise performance with the least possible fatigue either by performing fewer sets, adding more recovery time between sets, changing the angle of the exercise, introducing/combine different muscle groups, or not aiming for muscle failure. Future research would also be necessary to find out the recovery time of a protocol such as the one carried out in this study where muscle failure is not reached, or also to look for active recovery strategies between protocols.

As limitations of the study, more protocols could have been carried out using the same intensity but varying the volume of the series and repetitions so as not to reach muscle failure and thus have been compared with the protocol carried out in the study. In addition, the study is performed with healthy and active young men. Therefore, this should be considered when extrapolating these results to other older populations as well as to women. In addition, when comparing participants with different levels of strength, perhaps a larger number of participants in the Medium and High RSR would have been necessary.

## 5. Conclusions

After implementing a strength exercise protocol to muscle failure (three sets at 70% of 1RM, with MNR and 2 min of rest between sets), MPV shows a certain recovery from minute 10 of rest, remaining until minute 15, despite the existence of certain residual fatigue. However, the blood lactate levels are still high (≈4.5 mmol·L^−1^ in minute 15 post-exercise); thus, although the capacity to reach a maximum MPV peak was recovered, the non-recovery of metabolic homeostasis could alter the neuromuscular capacity to maintain these strength levels to reproduce again another exercise protocol involving the same muscle groups. Such tendency in the neuromuscular and metabolic recovery curves seems to be very similar regardless of the strength level of the participants.

## Figures and Tables

**Table 1:** Data related to MPV at 1 m·s^−1^ and blood lactate concentrations before and after the bench press exercise protocol with a load of 70% MPV obtained in the 1RM test for each individual.

Variable	Level of Strength	Pre-Exercise (M ± SD, 95% CI, CV)	0 min Post Exercise (M ± SD, 95% CI, CV)	5 min Post Exercise (M ± SD, 95% CI, CV)	10 min Post Exercise (M ± SD, 95% CI, CV)	15 min Post Exercise (M ± SD, 95% CI, CV)	p Time η_p_^2^ SP	p Group η_p_^2^ SP	p Group × Time η_p_^2^ SP
MPV at 1 m·s^−1^ (m·s^−1^)	High RSR (n = 11)	0.97 ± 0.07 † 0.92–1.01 7.2%	0.68 ± 0.13 ‡ 0.59–0.76 19.1%	0.87 ± 0.13 0.80–0.94 14.9%	0.92± 0.08 0.85–0.98 8.7%	0.91 ± 0.10 0.84–0.98 11%	<0.001 *	0.791	0.503
	Medium RSR (n = 14)	1.00 ± 0.08 * 0.96–1.03 8%	0.67 ± 0.15 ‡ 0.60– 0.74 22.4%	0.85 ± 0.13 £ 0.79–0.91 15.3%	0.92 ± 0.12 0.86–0.97 13%	0.91 ± 0.13 0.84–0.97 14.3%	0.793	0.011	0.042
	Low RSR (n = 19)	1.00 ± 0.07 * 0.96–1.03 7%	0.65 ± 0.12 ‡ 0.59–0.71 18.5%	0.83 ± 0.11 # 0.77–0.88 13.3%	0.87 ± 0.11 0.82–0.92 12.6%	0.89 ± 0.12 0.84–0.95 13.5%	1.000	0.085	0.362
Blood lactate (mmol·L^−1^)	High RSR (n = 11)	1.3 ± 0.3 * 1.1–1.6 20.6%	8.0 ± 2.5 & ¥ 6.9–9.1 30.7%	7.2 ± 1.7 $ 6.3–8.0 23.7%	5.9 ± 1.4 ¶ ¥ 5.2–6.6 24%	4.5 ± 1.0 3.8–5.2 21.4%	<0.001 *	0.030 *	0.008 *
	Medium RSR (n = 14)	1.5 ± 0.6 * 1.2–1.7 39%	7.5 ± 1.7 ‡ 6.6–8.5 22.3%	6.5 ± 1.2 $ 5.8–7.2 17.8%	5.7 ± 1.3 ¶ 5.01–6.3 22.7%	4.5 ± 1.1 3.9–5.1 24.2%	0.859	0.158	0.135
	Low RSR (n = 19)	1.5 ± 0.4 * 1.3–1.7 25.5%	6.1 ± 1.5 & 5.2–6.9 24.1%	5.9 ± 1.3 $ 5.3–6.5 22.4%	4.7 ± 1.0 4.2–5.3 20.8%	4.2 ± 1.2 3.7–4.7 28.1%	1.000	0.664	0.887

MPV = mean propulsive velocity; RSR = relative strength ratio, defined as 1RM value divided by body mass; M = mean ± SD = standard deviation; CI = confidence intervals; CV = coefficient of variation. η_p_^2^ = partial eta-squared; SP = statistical power. * = significant difference between pre-exercise and all post-exercise (*p* < 0.05). † = significant difference of pre-exercise with minute 0 and 5 post-exercise (*p* < 0.05). ‡ = significant difference of minute 0 post-exercise with minute 5, 10 and 15 post-exercise (*p* < 0.05). £ = significant difference between minute 5 and 10 post-exercise (*p* < 0.05). # = significant difference between minute 5 and 15 post-exercise (*p* < 0.05). & = significant difference of minute 0 post-exercise with minute 10 and 15 post-exercise (*p* < 0.05). $ = significant difference of minute 5 post-exercise with minute 10 and 15 post-exercise (*p* < 0.05). ¶ = significant difference between minute 10- and 15-min post-exercise (*p* < 0.05). ¥ = significant difference between High RSR and Low RSR (*p* < 0.05).

## Data Availability

Not applicable.
